# *In silico *identification of conserved microRNAs in large number of diverse plant species

**DOI:** 10.1186/1471-2229-8-37

**Published:** 2008-04-16

**Authors:** Ramanjulu Sunkar, Guru Jagadeeswaran

**Affiliations:** 1 Department of Biochemistry and Molecular Biology, Oklahoma State University, Stillwater, OK 74078, USA

## Abstract

**Background:**

MicroRNAs (miRNAs) are recently discovered small non-coding RNAs that play pivotal roles in gene expression, specifically at the post-transcriptional level in plants and animals. Identification of miRNAs in large number of diverse plant species is important to understand the evolution of miRNAs and miRNA-targeted gene regulations. Now-a-days, publicly available databases play a central role in the in-silico biology. Because, at least ~21 miRNA families are conserved in higher plants, a homology based search using these databases can help identify orthologs or paralogs in plants.

**Results:**

We searched all publicly available nucleotide databases of genome survey sequences (GSS), high-throughput genomics sequences (HTGS), expressed sequenced tags (ESTs) and nonredundant (NR) nucleotides and identified 682 miRNAs in 155 diverse plant species. We found more than 15 conserved miRNA families in 11 plant species, 10 to14 families in 10 plant species and 5 to 9 families in 29 plant species. Nineteen conserved miRNA families were identified in important model legumes such as *Medicago*, *Lotus *and soybean. Five miRNA families – miR319, miR156/157, miR169, miR165/166 and miR394 – were found in 51, 45, 41, 40 and 40 diverse plant species, respectively. miR403 homologs were found in 16 dicots, whereas miR437 and miR444 homologs, as well as the miR396d/e variant of the miR396 family, were found only in monocots, thus providing large-scale authenticity for the dicot- and monocot-specific miRNAs. Furthermore, we provide computational and/or experimental evidence for the conservation of 6 newly found Arabidopsis miRNA homologs (miR158, miR391, miR824, miR825, miR827 and miR840) and 2 small RNAs (small-85 and small-87) in *Brassica spp*.

**Conclusion:**

Using all publicly available nucleotide databases, 682 miRNAs were identified in 155 diverse plant species. By combining the expression analysis with the computational approach, we found that 6 miRNAs and 2 small RNAs that have been identified only in Arabidopsis thus far, are also conserved in *Brassica spp*. These findings will be useful for tracing the evolution of small RNAs by examining their expression in common ancestors of the *Arabidopsis*-*Brassica *lineage.

## Background

Cytoplasmic control of mRNA degradation and translation is one of the important strategies of eukaryotic gene expression programs. Recently discovered miRNAs are important regulators of gene expression at the post-transcriptional level. In plants, miRNA genes are transcribed by RNA polymerase II, and primary miRNAs transcripts are subsequently capped, spliced and poly-adenylated [1-4]. Plant miRNA processing appears to be confined to the nucleus, and only mature miRNAs are exported to cytoplasm [2]. In plants, DCL1 processes primary miRNA transcripts into an miRNA-miRNA* duplex, with 2-nt overhangs at the 3' end [2]. Arabidopsis hyponastic leaves (HYL1), a double-stranded RNA-binding domain (dsRBD)-containing protein, and SERRATE, a C2H2 zinc finger protein, assists DCL1 in releasing the miRNA duplex [5-7]. Then HEN1, a methyl transferase, adds methyl groups to the 3' ends of the duplex and stabilizes the miRNA duplex [8]. The miRNA duplex is then exported into the cytoplasm by HASTY, the plant ortholog of exportin 5 [9,10]. Only the active miRNA strand of the duplex, but not the passenger strand (miRNA*) is incorporated into the RNA-induced silencing complex (RISC). Guided by miRNA present in the RISC, the complex can recognize the target transcript and prevent protein production by degradation or translational repression [1,10-13].

In plants, miRNAs are implicated in diverse aspects of plant growth and development, including leaf morphology and polarity, lateral root formation, hormone signaling, transition from juvenile to adult vegetative phase and vegetative to flowering phase, flowering time, floral organ identity and reproduction [13,14]. A role of miRNAs in plant stress responses was also evident from recent studies. Several miRNAs are regulated in response to diverse stress conditions, which suggests that miRNA-directed post-transcriptional regulation of their respective target genes is important to cope with the stress [13,15-20].

Because miRNAs have emerged as vital components of post-transcriptional regulation of gene expression important for plant growth and development, as well as plant stress responses, identifying conserved miRNA homologs in as many plant species as possible is important. Computational approaches are successful in identifying conserved miRNAs in many plants and animals, but they require knowledge of the complete genome sequence, which is unavailable for most plant species. However, large genomic fragmented data in the form of genome survey sequences (GSSs), high-throughput genomics sequences (HTGSs) and nonredundant nucleotides (NRs), as well as expressed sequence tags (ESTs), are available for several plant species and can be used for identification of conserved miRNAs. GSS and HTGS of GeneBank represent only short stretches of genomic sequence but can still provide a broader sampling of unfinished genomes. The NR database contains finished genomic sequences and cDNAs. Previously Zhang et al. [21] identified conserved miRNAs in plants using ESTs alone.

Here, we used the available GSS, HTGS, and NR repositories and ESTs to identify a large number of conserved miRNA families in diverse plant species. Using BLAST searches for miRNA homologs coupled with secondary structure predictions with precursor sequences, we identified 682 miRNAs in 155 diverse plant species. Nineteen miRNA families were found in 3 legumes, *Medicago truncatula*, *Lotus japonicus *and *Glycine max*. Additionally, 6 miRNAs, previously thought to be Arabidopsis specific, are expressed in *Brassica spp*., which indicates that these miRNAs evolved recently in the *Arabidopsis*-*Brassica *clade and gives valuable information to trace their evolution.

## Results

### Identification of conserved plant miRNAs in 155 plant species

The basis for computational identification of miRNAs is the conserved, mature miRNA sequence coupled with the predictable secondary structure of miRNA surrounding sequences [22]. We used NCBI BLASTN to find miRNA sequences (orthologs/paralogs) matching at least 18 nt and leaving 3 nt for possible sequence variations in different plant species. To identify miRNA homologs in diverse plant species, the whole set of Arabidopsis and rice mature miRNA sequences from the miRBase (see Availability and requirements for URL)were used in BLAST searches against publicly available GSS, HTGS, EST and NR databases. The miRNA precursor sequences containing the miRNA sequences were extracted from the respective databases and used for fold-back structure predictions with use of mfold [23]. miRNAs are derived from either the 5' or 3' arm of the hairpin structure, which is also conserved across diverse plant species. To confirm this feature, the hairpin structures were compared with the previously reported miRNA hairpin structures. This search resulted in identification of miRNAs in 155 diverse plant species. Specifically, we found >15 miRNA families in 11 plant species, 10 to14 families in 10 plant species and 5 to 9 families in 29 plant species. Our survey also identified relatively more conserved miRNA families in some of the plant species. For instance, we found 23 miRNA families in maize, 19 in sorghum, 15 in wheat, and 14 in *Citrus sps*. Other notable miRNA families were found in some important plant species: 12 in grapes, 11 in tomato, 10 in sugarcane and 7 in potato. We also found five families (miR159, miR160, miR164, miR166 and miR168) conserved in gymnosperms and two (miR396 and miR408) in *Selaginella*.

Interestingly, miR319, miR156/157, miR169, miR165/166 and miR394 homologs were found in 51, 45, 41, 40 and 40 diverse plant species, respectively (Table 1 and see Additional file 1). Six families (miR159, miR160, miR167, miR170/171, miR396 and miR399) were found in 30–39 diverse plant species (Table 1). Similarly, seven families (miR164, miR168, miR172, miR393, miR395, miR398 and miR408) were found in 20–29 diverse plant species. The families, miR162, miR390, miR397, miR403 and miR437 were found in 10–19 diverse plant species.

**Table 1:** Diverse plant species with identified conserved miRNA families.

**miRNA family**	**Plant species**
miR156/157	*Arachis hypogea, Boechera stricta, Brassica napus, Brassica oleracea, Brassica rapa, Bruguiera gymnorhiza, Citrus × paradisi × Poncirus trifoliate, Euphorbia esula, Fragaria vesca, Gossypium hirsutum, Gossypium raimondii, Glycine max, Helianthus annuus, Ipomoea nil, Lycopersicon esculentum, Lactuca sativa, Lotus japonicus, Malus × domestica, Medicago truncatula, Nicotiana tabacum, Oryza australiensis, Oryza brachyanth, Oryza punctata, Oryza ridleyi, Oryza rufipogon, Oryza sativa, Populus trichocarpa, Poncirus trifoliata, Prunus persica, Solanum tuberosum Sorghum bicolor, Triticum aestivum, Zea mays*
miR158	*Brassica oleracea, Brassica rapa,*
miR159	*Boechera stricta, Brassica oleracea Brassica rapa, Euphorbia esula, Festuca arundinacea, Fragaria vesca, Glycine max, Hordeum vulgare, Oryza coarctata, Lactuca saligna, Lactuca serriola, Liriodendron tulipifera Lotus japonicus, Lycopersicon esculentum, Manihot esculenta, Medicago truncatula, Malus × domestica, Oryza alta, Oryza coarctata, Oryza ridleyi, Oryza rufipogon, Oryza sativa, Phaseolus vulgaris, Picea glauca, Pinus taeda, Populus deltoids, Populus tremula, Populus trichocarpa, Sorghum bicolor, Triticum aestivum, Vitis vinifera, Zea mays*
miR160	*Aquilegia formosa *× *Aquilegia pubescens, Beta vulgaris, Brassica rapa, Citrus sinensis, Citrus × paradisi *× *Poncirus trifoliate, Euphorbia esula, Festuca arundinacea, Gossypium hirsutum, Gerbera hybrida, Gossypium raimondii, Glycine max, Hordeum vulgare, Lotus japonicus, Lycopersicon esculentum, Manihot esculenta, Medicago truncatula, Malus × domestica, Oryza brachyantha, Oryza coarctata, Oryza glaberrima, Oryza ridleyi, Oryza sativa, Picea glauca, Picea engelmannii *× *Picea glauca, Populus trichocarpa, Populus trichocarpa *× *Populus deltoides, Prunus persica, Saccharum officinarum, Sorghum bicolor, Solanum tuberosum, Triticum aestivum, Vitis vinifera, Zea mays*
miR162	*Brassica oleracea, Euphorbia esula, Gossypium hirsutum, Gerbera hybrida, Glycine max, Helianthus petiolaris, Lactuca perennis, Lactuca saligna, Lactuca sativa, Lycopersicon esculentum, Medicago truncatula, Oryza sativa, Vitis vinifera, Zea mays*
miR164	*Brassica oleracea, Brassica rapa, Citrus sinensis, Gossypium hirsutum, Lotus japonicus, Medicago truncatula, Oryza australiensis, Oryza coarctata, Oryza glaberrima, Oryza granulate, Oryza minuta, Oryza punctata, Oryza rufipogon, Oryza sativa, Picea glauca, Populus trichocarpa, Populus tremula *× *Populus tremuloides, Poncirus trifoliate, Sorghum bicolor, Triticum aestivum, Zea mays*
miR165/166	*Aquilegia formosa *× *Aquilegia pubescens, Arachis hypogea, Brassica rapa, Brassica oleracea, Citrus sinensis, Citrus × paradisi *× *Poncirus trifoliate, Euphorbia esula, Gossypium hirsutum, Helianthus petiolaris, Hordeum vulgare, Lotus japonicus, Lycopersicon esculentum, Medicago truncatula, Malus × domestica, Oryza alta, Oryza brachyantha, Oryza coarctata, Oryza glaberrima. Oryza minuta, Oryza punctata, Oryza ridleyi, Oryza rufipogon, Oryza sativa, Phaseolus vulgaris, Pinus taeda, Populus deltoids, Populus trichocarpa, Sorghum bicolor, Vitis vinifera, Zea mays*
miR167	*Arachis hypogea, Brassica napus, Brassica oleracea, Brassica rapa, Citrus clementina, Fragaria vesca, Glycine max, Helianthus annuus, Ipomoea nil, Lotus japonicus, Medicago truncatula, Malus × domestica, Nicotiana tabacum, Oryza alta, Oryza australiensis, Oryza brachyantha, Oryza coarctata, Oryza granulate, Oryza minuta, Oryza nivara, Oryza punctata, Oryza ridleyi, Oryza rufipogon, Oryza sativa, Populus tremula, Populus trichocarpa *× *Populus deltoides, Populus tremula *× *Populus tremuloides, Saccharum officinarum, Sorghum bicolor, Triticum aestivum, Vitis vinifera, Zea mays*
miR168	*Beta vulgaris, Brassica napus, Brassica rapa, Citrus clementina, Glycine max, Ipomoea nil, Lotus japonicus, Malus × domestica, Picea glauca, Populus tremula, Populus trichocarpa, Vitis vinifera,*
miR169	*Brassica napus, Brassica oleracea, Brassica rapa, Carica papaya, Citrus sinensis, Euphorbia esula, Festuca arundinacea, Gossypium hirsutum, Glycine max, Helianthus petiolaris, Ipomoea nil, Lactuca sativa, Lactuca serriola, Lotus japonicus, Lycopersicon esculentum, Medicago truncatula, Oryza alta, Oryza australiensis, Oryza brachyantha, Oryza coarctata, Oryza granulate, Oryza minuta, Oryza nivara, Oryza officinalis, Oryza punctata, Oryza ridleyi, Oryza sativa, Populus tremula, Populus trichocarpa, Populus trichocarpa *× *Populus deltoides, Saccharum officinarum, Sorghum bicolor, Vitis vinifera, Zea mays*
miR170/171	*Aquilegia formosa *× *Aquilegia pubescens, Boechera stricta, Brachypodium distachyon, Brassica napus, Brassica oleracea, Brassica rapa, Carica papaya, Citrus sinensis, Euphorbia esula, Festuca arundinacea, Gossypium hirsutum, Hordeum vulgare, Lactuca perennis, Lactuca saligna, Lactuca sativa, Lotus japonicus, Lycopersicon esculentum, Malus × domestica, Medicago truncatula, Nicotiana tabacum, Oryza alta, Oryza brachyantha, Oryza coarctata, Oryza glaberrima, Oryza granulate, Oryza minuta, Oryza nivara, Oryza officinalis, Oryza punctata, Oryza ridleyi, Oryza rufipogon, Oryza sativa, Populus trichocarpa, Phaseolus vulgaris, Picea glauca, Pinus taeda, Prunus persica, Sorghum bicolor, Triticum aestivum, Vitis vinifera, Zea mays*
miR172	*Boechera stricta, Brassica oleracea, Brassica rapa, Citrus sinensis, Gossypium raimondii, Glycine max, Lactuca saligna, Lactuca sativa, Lotus japonicus, Lycopersicon esculentum, Manihot esculenta, Medicago truncatula, Malus × domestica, Oryza brachyantha, Oryza coarctata, Oryza sativa, Populus trichocarpa, Populus trichocarpa *× *Populus deltoides, Sorghum bicolor, Solanum tuberosum, Vitis vinifera, Zea mays*
miR319	*Brassica napus, Brassica oleracea, Brassica rapa, Glycine max, Ipomoea nil, Liriodendron tulipifera, Lotus japonicus, Lycopersicon esculentum, Medicago truncatula, Oryza glaberrima, Oryza minuta, Oryza punctata, Oryza rufipogon, Oryza sativa, Phaseolus vulgaris, Populus trichocarpa, Populus tremula *× *Populus tremuloides, Poncirus trifoliate, Saccharum officinarum, Sorghum bicolor, Triticum aestivum, Vitis vinifera, Zea mays*
miR390	*Boechera stricta, Brassica rapa, Citrus sinensis, Gossypium hirsutum, Lotus japonicus, Medicago truncatula, Oryza brachyantha, Oryza coarctata, Oryza granulate*
miR391	*Brassica oleracea,*
miR393	*Brassica napus, Brassica oleracea, Brassica rapa, Gossypium hirsutum, Gerbera hybrida, Glycine max, Lotus japonicus, Medicago truncatula, Malus × domestica, Oryza minuta, Oryza sativa, Picea glauca, Picea engelmannii *× *Picea glauca, Pinus taeda, Populus trichocarpa *× *Populus deltoides, Sorghum bicolor, Zea mays*
miR394	*Aquilegia formosa *× *Aquilegia pubescens, Brassica napus, Brassica oleracea, Brassica rapa, Citrus clementina, Citrus sinensis, Euphorbia esula, Gossypium hirsutum, Gossypium raimondii, Glycine max, Helianthus annuus, Hordeum vulgare, Ipomoea nil, Lactuca sativa, Lactuca serriola, Liriodendron tulipifera, Medicago truncatula, Malus × domestica, Oryza coarctata, Oryza glaberrima, Oryza minuta, Oryza sativa, Picea glauca, Picea engelmannii *× *Picea glauca, Populus deltoids, Populus tremula, Populus trichocarpa, Poncirus trifoliate, Prunus persica, Saccharum officinarum, Sorghum bicolor, Solanum tuberosum, Triticum aestivum, Vitis vinifera, Zea mays*
miR395	*Boechera stricta, Brassica oleracea, Brassica rapa, Gossypium hirsutum, Glycine max, Lotus japonicus, Medicago truncatula, Oryza alta, Oryza australiensis, Oryza coarctata, Oryza rufipogon, Oryza sativa, Populus trichocarpa, Saccharum officinarum, Sorghum bicolor, Triticum aestivum, Vitis vinifera, Zea mays*
miR396	*Beta vulgaris, Brassica napus, Brassica oleracea, Bruguiera gymnorhiza, Citrus clementina, Festuca arundinacea, Gossypium hirsutum, Glycine max, Hordeum vulgare, Lactuca sativa, Lotus japonicus, Medicago truncatula, Oryza coarctata, Oryza glaberrima, Oryza minuta, Oryza officinalis, Oryza sativa, Pinus taeda, Populus trichocarpa, Populus trichocarpa *× *Populus deltoides, Populus tremula *× *Populus tremuloides, Prunus persica, Saccharum officinarum, Sorghum bicolor, Solanum tuberosum, Zea mays*
miR397	*Brassica oleracea, Brassica rapa, Nicotiana tabacum, Oryza alta, Oryza brachyantha, Oryza coarctata, Oryza minuta, Oryza nivara, Oryza rufipogon, Oryza sativa, Populus tremula, Populus trichocarpa *× *Populus deltoides, Zea mays*
miR398	*Brassica oleracea, Brassica rapa, Gossypium hirsutum, Gossypium raimondii, Glycine max, Helianthus petiolaris, Lactuca perennis, Lactuca saligna, Lactuca serriola, Lotus japonicus, Medicago truncatula, Oryza sativa, Picea glauca, Poncirus trifoliate, Sorghum bicolor, Triticum aestivum, Zea mays*
miR399	*Boechera stricta, Brassica napus, Brassica oleracea, Brassica rapa, Carica papaya, Citrus sinensis, Fragaria vesca, Gossypium hirsutum, Lactuca sativa, Lotus japonicus, Lycopersicon esculentum, Medicago truncatula, Oryza australiensis, Oryza brachyantha, Oryza coarctata, Oryza glaberrima, Oryza nivara, Oryza punctata, Oryza rufipogon, Oryza sativa, Populus tremula, Populus tremula *× *Populus tremuloides, Prunus persica, Sorghum bicolor, Solanum tuberosum, Triticum aestivum, Vitis vinifera, Zea mays*
miR403	*Arabidopsis thaliana, Boechera stricta, Brassica napus, Brassica oleracea, Brassica rapa, Carica papaya, Helianthus annuus, Helianthus argophyllus, Helianthus petiolaris, Lycopersicon esculentum, Malus × domestica, Populus trichocarpa, Populus tremula *× *Populus tremuloides, Poncirus trifoliate, Solanum tuberosum, Taraxacum officinale*
miR408	*Brachypodium distachyon, Brassica napus, Brassica rapa, Bruguiera gymnorhiza, Citrus × paradisi *× *Poncirus trifoliate, Euphorbia esula, Fragaria vesca, Glycine max, Lotus japonicus, Medicago truncatula, Oryza minuta, Oryza officinalis, Oryza sativa, Pinus taeda, Populus trichocarpa, Poncirus trifoliate, Prunus persica, Saccharum officinarum, Sorghum bicolor, Triticum aestivum, Zea mays*
miR437	*Oryza coarctata, Oryza granulate, Oryza minuta, Oryza punctata, Oryza sativa, Saccharum officinarum, Sorghum bicolor, Triticum aestivum, Zea mays*
miR444	*Brachypodium distachyon, Hordeum vulgare, Oryza minuta, Oryza officinalis, Oryza sativa, Saccharum officinarum, Sorghum bicolor, Panicum virgatum, Triticum aestivum, Zea mays*

In dicots, leguminous plants form an important source of human and animal dietary needs second only to cereal plants. Molecular tools, including genomics, are being used to rapidly develop *Medicago truncatula*, *Lotus japonicus *and *Glycine max *as model legumes to pursue a number of important biological questions unique to these plants. However, only a few miRNAs from these important legumes have been recorded in the miRNA registry. With the exception of miR397 and miR403, our survey has identified the remaining 19 conserved miRNA families in legumes (Table 1 and see Additional file 1). Among the ~21 miRNA families conserved between dicots and monocots, miR319 homologs were found in the largest number (51) of plant species, whereas miR397 homologs were found in the least number (14) of plant species. By searching all gene bank sources, we obtained a wider coverage, both in terms of miRNA families and number of diverse plant species.

On the basis of mature miRNA sequence similarity, these miRNAs were grouped into families, with members often varying by 1 to 2 nt. Here, we found 16 new miRNAs belonging to 11 miRNA families in diverse plant species. This includes one new member for each of the families, miR158, miR159, miR160, miR172, miR390, miR395 and miR408. We also identified two new members belonging to miR319, miR398 and miR403 families and three new members belonging to miR169 (Table 1).

Zhang et al. [21] classified the miRNAs as highly, moderately or lowly conserved, based on the number of plants in which each family of miRNA is predicted, although the number of ESTs available for different plant species varies highly. Accordingly, miR395, miR399, miR403 and miR408 families were classified as lowly conserved [21]. Zhang et al. retrieved miR395 and miR399 homologs from nine and eight plant species, respectively, which formed the basis for the authors' categorization of the families as being lowly conserved [21]. miR395 and miR399 are specifically up-regulated in response to low nutrient conditions. miR399 is induced under low phosphate conditions [16,18,24,25], whereas miR395 is induced in response to low-sulfate conditions [15]. Thus the representation of primary miR395 and miR399 transcripts in the ESTs generated from untreated plants is highly unlikely. By contrast, using GSS, HTGs, EST and NR databases, we found miR399 and miR395 homologs in as many as 28 and 18 diverse plant species, respectively. In fact, with use of GSS alone, miR395 and miR399 homologs were retrieved from 9 and 11 diverse plant species, respectively (Table 1). These results suggest that these two miRNA families are not lowly conserved miRNAs, as previously considered.

miR408 was cloned from Arabidopsis and rice [3,26]. By searching the EST database alone, miR408 homologs were found in nine plant species. As a result, Zhang et al. [21] classified miR408 as one of the lowly conserved miRNAs. In this study, we found miR408 homologs in 23 diverse plant species, including *Selaginella *(Table 1). Thus, miR408 is one of the deeply conserved miRNAs. miR408 has been shown to guide cleavage of plantacyanin, its target transcript in rice [3]. Also in a recent report, miR408 was found to be expressed in *Selaginella *and to target a conserved plantacyanin transcript [27]. The deep conservation of miR408 across the plant kingdom indicates that the regulation of plantacyanin transcript levels has been preserved for a long time. Similarly, we found miR403 homologs in 16 plant species (Table 1); therefore miR403 is not a lowly conserved miRNA as classified by Zhang et al. [21]. Together, these findings indicate that the classification of miRNAs as highly, moderately and lowly conserved miRNAs on the basis of available ESTs alone may not reflect the true depth of conservation.

### Dicot- and monocot-specific miRNAs

**Figure 1:**
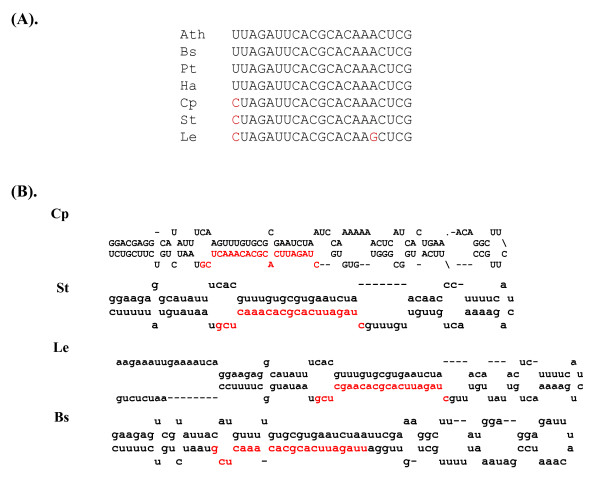
**miR403 in several dicotyledonous plants.** (A) miR403 homologs in Arabidopsis, *Populus trichocorpa*, *Solanum tuberosum*, *Carica papaya, Lycopersicum esculentum *and *Helianthus annus*. (B) Predicted fold-back structures using miR403 precursor sequences.

miR403 was initially identified in Arabidopsis and later found in *Populus trichocorpa *[4,26,28,29]. In a previous report, miR403 was considered a dicot-specific miRNA because its homologs were not found in rice. In the present study, we found miR403 homologs in 16 dicotyledonous plants, including *Populus*, papaya, tomato, potato, sunflower, and *Brassica spp *(Table 1 and Figure 1A,B), but not in monocotyledonous plants. The identification of miR403 homologs in other dicots revealed two new members of this miRNA family. As compared to the Arabidopsis mature miR403 sequence, miR403 differed at the 5' most nucleotide in *Papaya *and potato and the 5' most 2 nt in tomato (Table 1). Thus, the miR403 family is represented by at least three members in dicots. The identification of miR403 in as many as 16 dicots provided large-scale authenticity for considering it a dicot-specific miRNA.

**Figure 2:**
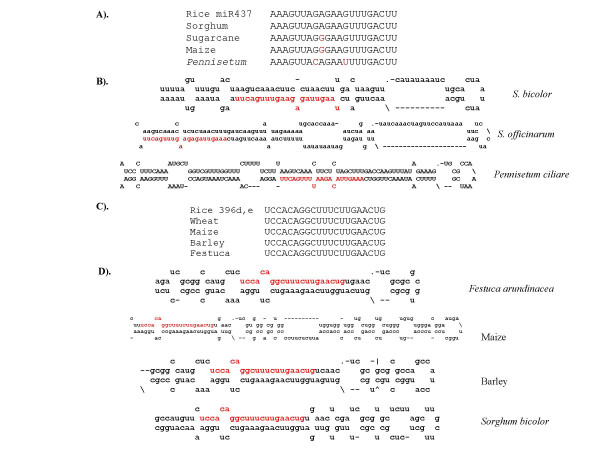
**Monocot-specific miRNAs.** A) miR437 homologs from rice, *Sorghum*, sugarcane, maize and *Pennisetum*. (B) Predicted fold-back structures for the miR437 precursor sequences from rice and *Pennisetum ciliare*. (C) miR396d,e homologs in rice, wheat, *Festuca arundancea*, barley and Maize. (D). Predicted fold-back structures for the miR396d/e precursor sequences from wheat (CJ776495), maize (EST DR802570), barley (EST AV925436, *Festuca arundinacea *(EST DT684101) and *Sorghum bicolor *(GSS).

Sequencing of rice small RNA libraries resulted in the identification of a few monocot-specific miRNAs [3]. Rice miR437 homologs found in maize, sorghum and sugarcane but not in Arabidopsis or *Populus *led to the suggestion that miR437 may be a monocot-specific miRNA [3]. In this study, we found additional evidence to support classifying miR437 as a monocot-specific miRNA, because miR437 homolog was recovered from *Pennisetum ciliare*, another monocot (Figure 2A and 2B). Similarly, miR444 has been reported as a monocot-specific miRNA [3]; its homologs were found in wheat, barley, sorghum, switchgrass, sugarcane, *Brachypodium distachyon, Oryza officinalis *and *Oryza minuta *(Table 1). Recently, five additional members of the miR444 family, all of which are conserved only in monocots were reported (30).

miR396 homologs were found to be deeply conserved [27]. miR396 in rice is represented by two variants with five loci (OsmiR396a,b,c and OsmiR396d,e) [3]. The mature miRNA sequence corresponding to OsmiR396a,b,c is conserved across dicots and monocots. The other variant, represented by OsmiR396d,e, differs from OsmiR396a,b,c by an additional nucleotide "G" between positions 8 and 9 [3]. Because the exact sequence of miR396d,e has not been found in the *Arabidopsis *or *Populus *genomes and its expression could not be detected in Arabidopsis, it was considered a monocot-specific version of the miR396 family [3]. Consistent with this suggestion, miR396d,e homologs were identified in five other monocots – *Sorghum bicolor*, maize, wheat, barley and *Festuca arundancea *– and a hairpin structure could be predicted for all of these miRNA precursors (Figure 2C). Thus, the identification of miR437, miR444 and the miR396d/e variant of the miR396 family in several monocots provided solid support for consideration of these miRNAs as being monocot specific.

### Arabidopsis-*Brassica *lineage-specific miRNAs

**Figure 3:**
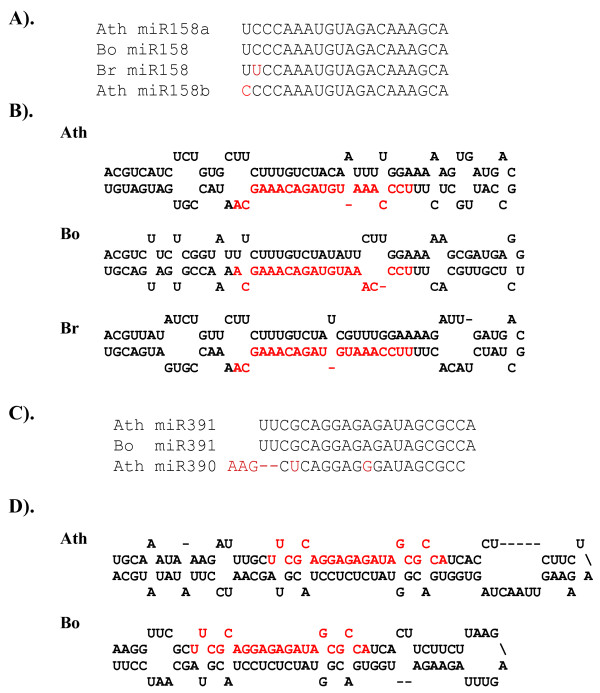
***Arabidopsis-Brassica***** lineage-specific miRNAs.** (A) miR158 homologs in Arabidopsis and *Brassica oleracea *and *Brassica rapa*. (B) Predicted fold-back structures with miR158 precursor sequences from *B. oleracea *and *B. rapa*. (C). miR391 homologs from Arabidopsis and *Brassica oleracea *aligned with Arabidopsis miR390. (D). Predicted fold-back structures using miR391 sequences from Arabidopsis and *Brassica oleracea*.

**Figure 4:**
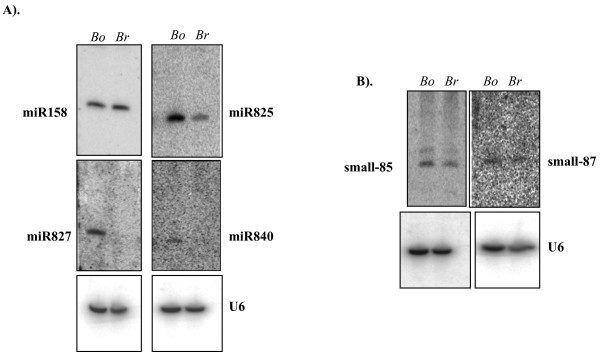
**Small RNA blot analysis of newly identified small RNAs in *Brassica*.** An amount of 20 μg of low-molecular-weight RNA used for northern analysis. Antisense oligonucleotide probes were designed for the Arabidopsis miRNAs to detect their expression in *Brassica oleracea *(Bo) and *Brassica rapa *(Br) seedlings. Radiolabeled antisense oligonucleotide probes were used for detection of miRNAs (A) or radiolabeled antisense LNA-probes for detection of small-85 and small-87 (B). Blots were re-probed with U6, which served as a loading control.

An initial experimental approach led to the identification of at least four non-conserved miRNAs in Arabidopsis. miR158 is one among them, and is represented by two loci (miR158a and miR158b) in Arabidopsis [31] and miR158 homologs are not computationally/experimentally evident either in rice [3,15,32] or in poplar [17]. Therefore, miR158 has been considered an Arabidopsis-specific miRNA. Here, we found computational evidence for the presence of miR158 homologs in two *Brassica *sps. (Figure 3A). Further, the mature miR158 sequence and the sequence that adopts the fold-back structure is highly conserved in *Brassica oleracea *and *Brassica rapa *(Figure 3B). miR158 in *B. rapa *differed from miR158 in Arabidopsis by 2 nt at the 5' end. Northern blot analysis with labeled miR158 antisense oligonucleotide revealed that miR158 is abundantly expressed in *B. oleracea *and *B. rapa *seedlings (Figure 4A).

miR391 is one of the recently identified miRNAs that has some sequence similarity with the miR390; therefore, Xie et al. [4] considered it a member of the miR390 family. Although miR390 is one of the broadly conserved miRNAs, the miR391 sequence has not been identified in plants other than Arabidopsis, which led to the hypothesis that miR391 is a non-conserved Arabidopsis-specific miRNA [4]. Our search revealed an miR391 homolog, and a fold-back structure could be predicted for the precursor sequence in *B. oleracea *(Figure 3C and 3D).

Recent deep sequencing of Arabidopsis small RNAs suggested that the Arabidopsis genome encodes more non-conserved miRNA families than conserved miRNA families [19,33,34]. These newly found Arabidopsis miRNAs are considered non-conserved because the orthologous sequences have not been found in the rice or *Populus *genomes [19,33,34]. The non-conserved plant miRNAs presumably emerged and dissipated in short evolutionary time scales [19,34]. High-throughput sequencing of small RNAs from species closely related to Arabidopsis would help define the lifespan of these transient miRNA genes [34]. Bioinformatic inspection of the conservation of these miRNAs in *Brassica *may not be completely informative at this time because of the lack of complete genome information and the search for these miRNA precursor sequences among ESTs has been unsuccessful. Because these newly found miRNAs have been recovered only in high-throughput sequencing suggests that their abundance is extremely low, and thus their representation in ESTs is unlikely. To examine whether any of these newly found miRNA homologs are expressed in *Brassica*, a close relative of Arabidopsis, we performed small RNA blot analysis using RNA isolated from two *Brassica spp*. (*B. oleracea *and *B. rapa*). To enhance the detection ability, we used low-molecular weight RNA isolated from 4-week old seedlings of *B. oleracea and B. rapa*. The expression of 10 of the newly found miRNAs (miR771, miR773, miR775, miR825, miR827, miR828, miR837, miR840, miR846 and miR848) was analyzed. We chose these miRNAs because they could be detected on small-RNA blot analysis in Arabidopsis and were relatively more abundant in the libraries than other newly found miRNAs in Arabidopsis [19,33,34]. Three of the miRNAs (miR825, miR827 and miR840) could be detected in one or both of the *Brassica spp*, although their expression levels varied greatly (Figure 4A). For instance, miR825, miR827 and miR840 were more abundant in *B. oleracea *than in *B. rapa *(Figure 4A). Surprisingly, we were unable to detect a signal for miR827 and miR840 in *B. rapa *(Figure 4A). Computational analysis revealed miR824 and miR828 homologs in *Brassica *(data not shown), although we were not successful in detecting a signal using a probe against miR828 in *Brassica *seedlings. miR828 appears to be specifically or abundantly expressed in siliques of Arabidopsis [34]. Recently, conserved miR824 homologs were found in 3 *Brassica spp*. [35].

Computational analysis revealed the conservation of miR158, miR391 and miR824 in *Brassica spp*, and our small RNA blot analysis confirmed the expression of miR827, miR825, and miR840 in at least one of the *Brassica spp *(Figures 3 and 4A). Thus, 6 of the miRNAs (miR158, miR391, miR824, miR825, miR827 and miR840), whose expression is not known outside Arabidopsis, are indeed conserved between Arabidopsis and *Brassica*.

Arabidopsis and rice are known to express a large number of non-conserved diverse small-interfering RNAs (siRNAs) [36-38]. The only exception to-date is that *trans*-acting siRNAs (Tas3a,b,c), a sub-class of siRNAs that are deeply conserved [39,40]. Recently, Lu et al. [33] found a few non-miRNA small RNAs in Arabidopsis. We used small-RNA blot analysis to test whether any of the three small RNAs (small-85, small-86 and small-87) are conserved between Arabidopsis and *Brassica*. Surprisingly, small-85 and small-87 could be detected in both *Brassica *species we tested (Figure 4B), which suggests that these two small RNAs are conserved between Arabidopsis and *Brassica *and represent lineage-specific small RNAs.

### Clusters of plant miRNAs

Clusters of miRNAs frequently found in animals are transcribed together as a polycistron [10,41-44]. Although miRNA clusters are not common in plants, a few miRNA families (miR395, miR399, miR169 and miR1219) have been found to exist as clusters [26,45-47]. Recently, two tandem miR156 homologs were reported in rice and maize [48,49]. Here, we identified an miR156 cluster in several other plant species: two tandem miR156 homologs located within 370 nt of the same orientation in the rice EST AK110797, two miR156 homologs separated by ~190 nt in the sugarcane EST CA294779, two miR156 homologs separated by 340 nt in the EST CL172990 of *Sorghum bicolor*, and two miR156 homologs separated by 301 nt in the maize EST CL985276. Additionally two very closely spaced miR156 homologs were found in a genomic clone of *Oryza granulata *(216 nt), *Oryza punctata *(370 nt). In comparing the syntenic regions among 3 cereals (i.e., rice, sorghum and maize), Wang et al. [49] suggested that two miR156 homologs in tandem arrangement are highly conserved among cereals. Interestingly, we found a similar arrangement of two tandem miR156 homologs separated by 590 nt in the EST CJ743424 of *Ipomea nil*, a dicotyledonous plant. These findings suggest that the tandem arrangement of two miR156 homologs is not restricted to cereals and seems to exist in diverse plant species that are distantly related.

We also found two tandem miR169 homologs in the same orientation and separated by 250 nt in the cotton genomic clone DX401397. Two miRNAs belonging to the miR169 family in cotton (46) and *Brassica napus *(49) have been recently reported. Because these homologs are close together argues against their origin from two different miR169 primary transcripts, although evidence for the expression of these two miR169 homologs in one transcript in the form of an EST is lacking. Additionally, miR169 homologs were found in clusters in *Lactuca sativa *(DY980357), *Populus tremula *(CK111070) and *Euphorbia esula *(DV142897) but not in Arabidopsis or rice. Thus, we show miRNA gene clustering for miR156 and miR169 loci in diverse plant species. The results suggest that at least four miRNA families (miR156, miR169, miR395 and miR1219) exist as miRNA clusters in plants.

## Discussion

Recent studies have established that miRNAs play critical roles in post-transcriptional gene expression in higher eukaryotes. Evidence for conservation of plant miRNAs has come from genomic and EST sequence data from diverse plants showing sequences containing miRNA hairpins as well as sequences homologous to the known or predicted Arabidopsis targets retaining miRNA complementary sites [15,21]. To date, ~21 miRNA families known to be conserved between dicots and monocots forms the basis for the identification of these miRNA families in diverse plant species by use of publicly available nucleotide databases. By searching these databases, we identified a total of 682 miRNAs in 155 different plant species. Our analysis yielded >15 conserved miRNA families in 11 plant species and 10 to14 conserved families in 10 plant species. We also identified relatively more conserved miRNA families (i.e., 23 in maize, 19 in *Sorghum*, 15 in wheat, 14 in *Citrus*, 12 in grapes, 11 in tomato, 10 in sugarcane and 7 in potato). At least five families (miR319, miR156/157, miR169, miR165/166 and miR394) were found in more than 40 plant species (Table 1). We found six families (miR159, miR160, miR167, miR170/171, miR396 and miR399) in 30–39 species; seven (miR164, miR168, miR172, miR393, miR395, miR398 and miR408) in 20–29 species; and five (miR162, miR390, miR397, miR403 and miR437) in 10–19 species (Table 1). Computational analysis coupled with expression analysis provided evidence for six of the newly found miRNAs as being conserved between Arabidopsis and *Brassica*. Additionally, some of the non-miRNA small RNAs (small-85 and small-87) found in Arabidopsis were also found in *Brassica *(Figure 4B). These findings provide the first large-scale identification of lineage-specific miRNAs and other small RNAs.

miR395 and miR399 are specifically induced under low-sulfate and low-phosphate conditions, respectively [15,16,18,24,25]. miR399 and miR395 homologs are in as many as 31 and 22 diverse plant species, respectively (Table 1). miR399 plays an important role in phosphate homeostasis [16,18]. Similarly, miR398 homologs were found in 22 plant species. The down-regulation of miR398 has been implicated in up-regulating Cu/Zn-superoxide dismutase 1 (*CSD1*) and 2 (*CSD2*)in Arabidopsis in response to oxidative stress conditions [13,20]. In contrast, miR398 is up-regulated in response to Cu^2+ ^limiting conditions [50]. miR398 induction is inversely correlated with the expression of *CSD1 *and *CSD2 *genes, thus maintaining Cu^2+ ^homeostasis and mobilizing the available Cu^2+ ^to more indispensable proteins such as plastocyanin [50]. miR393 and its target gene TIR1 are conserved [15,26]. A role for miR393 in Arabidopsis disease resistance has been shown recently [51]. Thus, we found several stress-responsive miRNA homologs – miR393, miR398, miR395 and miR399 – highly conserved in diverse monocots and dicots, which suggests that these miRNA-guided target gene regulations have been well preserved, possibly because they are important for plant stress tolerance [13].

Recent deep sequencing of plant small RNA libraries clearly demonstrated that plants express more non-conserved than conserved miRNAs [19,30,34]. The non-conserved miRNAs presumably emerged and dissipated in short evolutionary time scales [19]. Such rapid emergence of new genes is likely facilitated by the small size and simple architecture of miRNA genes derived from their targets [52], although whether such mechanisms are relevant for most newly emergent miRNAs [19,34] is unclear. Small-RNA blot analysis for 10 of the newly found miRNAs confirmed that 3 are expressed in *Brassica *seedlings. Most of the newly found non-conserved miRNAs in Arabidopsis are abundantly expressed in inflorescence [33,34,36], but we did not test this expression. Thus, the remaining seven miRNAs not detected in *Brassica *seedlings need further study. The absence of expression of some of the new miRNAs in *Brassica *could be due to their loss in *Brassica*, or they recently evolved in Arabidopsis after the divergence.

The existence of miRNAs and Tas3-derived tasiRNAs in plants is well known [39,40]. Interestingly, in the present study, we found two small RNAs (non-miRNAs and non-tasiRNAs) conserved between Arabidopsis and *Brassica*. Small-85, has been recently identified [33] and is derived from a long perfect fold-back structure that is reminiscent of siRNAs derived from dsRNA. Small-85 accumulation was dependent on all four of the dicers in Arabidopsis [33]. It disappeared only in a quadruple *dcl *(dcl-1,2,3,4) mutant but accumulated alone in *dcl1 *or in a triple mutant [33]. Small-85 is derived from the SRK gene that is capable of adopting a fold-back structure, and its expression is not dependent on RDR2 [32].

Loss of self-incompatibility in *Arabidopsis thaliana *and *Brassica *is thought to be due to inactivation of a self-incompatibility (SI) system that involves *SRK *and *SCR *genes. In the *Brassica *SI system, genes encoding for SI specificity in pistil (SRK) and pollen (SCR) are thought to be preserved because of rare or no recombination, and disruption of this structure would lead to loss of SI. Loss of the SI system in *A. thaliana *Columbia-0 (Col-0) was attributed to non-functional *SRK *and *SCR *genes [53]. Lu et al. [33] hypothesized a role for small-85 in loss of SRK function in *A. thaliana *with its accumulation. Here, we showed that the Arabidopsis small-85 probe can detect a strong signal at the expected size range in two *Brassica *species, which indicates that small-85 RNA also accumulates in *Brassica *seedlings. Further studies are required to clarify the role of this small RNA in self-incompatibility. The expression of several *SRK *genes from self-compatible plant species in vegetative tissues suggests that *SRKs *may play a developmental role. Similarly, the detection of small-85 in *Brassica *seedlings also suggests its role in development in *Brassica*.

Until now, only miR395 homologs were found to exist as clusters in Arabidopsis and rice [45]. Some of these clusters are co-transcribed because they were found in ESTs of rice [45]. Similarly, the clustered organization of miR1219 in *Physcometrella *was recently reported [47]. Although miR399 homologs in Arabidopsis and rice were found to be closely spaced [26], their expression in one transcript is unknown. Our analysis indicated that along with the well-documented clustered organization of miR395, miR156 and miR169 also exist as clusters in several plant species. These observations suggest that the tandem duplications are the cause for such an organization. Retention of tandem duplications may be due dosage response in some plants. Gene duplication is estimated to occur at a higher rate in eukaryotic genomes in general [54] and in flowering plants in particular [55,56].

Although several similar attempts were made earlier (21, 28, 46, 57, 58), largely these studies used either single plant species (for example, cotton or *Brassica sps*) or single nucleotide repository (ESTs). In this study, we used all nucleotide repositories and considered all plant species. Furthermore, earlier reports (21, 28, 46, 57, 58) included small RNAs that were initially identified as miRNAs but turned out to be siRNAs (e.g., miR404-miR407 in Arabidopsis and miR439, miR442 and miR445 in rice). Here, we used a conservative approach and considered only miRNAs that are confidently annotated for the identification of homologs in diverse plant species.

The identification of conserved miRNAs by searching all available nucleotide databases allows for wider and better coverage of diverse plant species than that with use of the EST database alone. Our discovery of some of the recently found Arabidopsis miRNAs conserved in *Brassica*, a close relative of Arabidopsis, will help in tracing the evolution of these miRNAs by analyzing their expression in common ancestors of *Brassica *and Arabidopsis. Arabidopsis and *B. oleracea *are closely related species that diverged from a common ancestor approximately 15–20 million years ago [59]. Because some miRNAs have been found in both Arabidopsis and *Brassica*, these miRNAs may be present in their ancestors. Expression analysis of the origin of Brassicacea (e.g., *Carica papaya*), at the base of the order Brassicales, or Cleomaceae, a sister to Brassicaceae, will provide close, intermediate and distant comparisons to trace the evolution of these miRNAs.

## Conclusion

Using all publicly available nucleotide databases, 682 miRNAs were identified in 155 diverse plant species. By combining the expression analysis with the computational approach we found that 6 miRNAs and 2 small RNAs that have been identified only in Arabidopsis thus far, are also conserved in *Brassica spp*. These findings will be useful for tracing the evolution of small RNAs by examining their expression in common ancestors of the *Arabidopsis*-*Brassica *lineage.

## Methods

### Blast search against NCBI gene repositories

All previously recorded miRNAs in Arabidopsis, rice, *Populus *and *Physcometrella *species were obtained from the miRBase (Release 10.0, August 2007), and we extracted the non-redundant miRNA sequences. We used these sequences for a BLASTN search of homologs in the GSS, HTGS, EST and NR databases. We adopted mature miRNA sequences matching at least 18 nt and leaving 0–3 nt for possible sequence variations in diverse plants. We used 4-nt variation cautiously and considered whether this 4-nt variant was also conserved in other plants. BLASTN parameters were essentially the same as described previously [21,46]. The parameters were expected values of 1000 and number of descriptions and alignments of 1000. The default word-match size between the query and database sequences was 7. If the matched sequence was shorter than the queried miRNA sequence, the aligned parts were manually compared to determine the number of matching nucleotides. Wherever available, precursor sequences of 620-nt were extracted (300-nt upstream and 300-nt downstream to the BLAST hits) and used for the hairpin structure predictions. For GSSs, we used the entire available sequence as an miRNA precursor sequence. These precursor sequences were retrieved and used for BLASTX analysis; we removed the protein coding sequences and retained only the non-protein sequences. Precursor sequences of these potential miRNA homologs underwent hairpin structure predictions by use of the RNA secondary-structure prediction software mfold [23]. We used a cutoff of less than six mismatches between the miRNA and miRNA* sequence in the other arm. Computational studies have reported that miRNA precursor sequences have significantly higher negative minimal folding free energies (MFEs) and minimal folding free energy indexes (MFEIs) than other non-coding RNAs or mRNAs [60]. As reported by Zhang et al. [60], we used an MEFI cutoff of 0.85. Finally, the hairpin structures were examined and compared with those of previously reported miRNAs for confirming the location of mature miRNA sequences within the hairpin. In brief, the following criteria were applied in designating the RNA sequence as an miRNA homolog: (1) an RNA sequence folding into an appropriate stem-loop hairpin secondary structure, (2) a mature miRNA sequence site in one arm of the hairpin structure, (3) miRNAs having less than six mismatches with the opposite miRNA* sequence in the other arm, (4) no loop or break in miRNA* sequences, (5) predicted secondary structures with higher MFEIs and negative MFEs, and (6) predicted mature miRNAs with no more than 3 nt substitutions as compared with *A. thaliana*, rice, *Populus *and *Physcometrella *mature miRNAs. These parameters fulfilled the criteria proposed by Ambros and co-workers [61].

### RNA gel blot analysis

Total RNA was isolated from four-week-old rice seedlings left untreated (control) or exposed to salt stress or drought stress with use of Trizol Reagent. Low-molecular-weight RNA was isolated from total RNA by use of PEG precipitation. An amount of 20 μg low-molecular-weight RNA was loaded per lane, resolved on a denaturing 15% polyacrylamide gel, and transferred electrophoretically to Hybond-N+ membranes (Amersham Biosciences, Buckinghamshire, UK). Membranes were UV cross-linked and baked for 2 h at 80°C. DNA oligonucleotides complementary to miRNA sequences were end labeled with γ-32P-ATP by use of T4 polynucleotide kinase (New England Biolabs). Membranes were prehybridized for at least 1 h and hybridized overnight with use of Perfect hybridization buffer (Sigma) at 38°C. Blots were washed three times (twice with 2 × SSC + 1% SDS and once with 1 × SSC + 0.5% SDS) at 50°C. The membranes were briefly air dried, then exposed to phosphorscreen, and images were acquired by scanning the films with use of a Typhoon. Two small-RNA sequences tested for their expression in *Brassica *were small-85 (small-85 CAAGACAATAATCTTCTCGGCTA) and small-87 (small-87 AAGAACATCCAAGGTGTTTGT) [32].

## Authors' contributions

RS designed the research, RS and GJ performed the research, RS wrote the paper.

## Availability and requirements

To identify miRNA homologs in diverse plant species, the whole set of Arabidopsis and rice mature miRNA sequences from the miRBase (Release 10.0, August 2007; ) were used in BLAST searches against publicly available GSS, HTGS, EST and NR databases.
